# Analysis of the quality of prenatal data of pregnant women attended at Healthcare Services in the city of São Paulo between 2012 and 2020

**DOI:** 10.1590/1980-549720230051

**Published:** 2023-11-13

**Authors:** Fernanda Ferreira Corrêa, Thaís Rangel Bousquet Carrilho, Eliana de Aquino Bonilha, Victor Nahuel Keller, Tarcisio Cantos de Melo, Gilberto Kac, Carmen Simone Grilo Diniz

**Affiliations:** I Universidade de São Paulo, School of Public Health – São Paulo (SP), Brazil.; II Universidade Federal do Rio de Janeiro, Josué de Castro Nutrition Institute, Nutritional Epidemiology Observatory – Rio de Janeiro (RJ), Brazil.; III Centro Universitário São Camilo – São Paulo (SP), Brazil.; IV Universidade de São Paulo, School of Public Health, Gender, Evidence and Health Study Group – São Paulo (SP), Brazil.

**Keywords:** Health care, Data systems, Pregnant women, Quality indicators, health care, Atenção à saúde, Sistemas de dados, Gestantes, Indicadores de qualidade em assistência à saúde

## Abstract

**Objective:**

To analyze the quality of data collected during prenatal care recorded in the Integrated Health Care Management System (SIGA) of the Municipal Department of Health of São Paulo from 2012 to 2020.

**Methods:**

Descriptive study using SIGA data and the variables: maternal height (cm), weight (kg) measured throughout pregnancy, gestational age at prenatal consultation, systolic (SBP) and diastolic (DBP) blood pressure (in mmHg), and body mass index (BMI) at the beginning of pregnancy (up to 8 weeks). Quality analysis was carried out by calculating the indicators: percentage of incompleteness and zero values of all variables studied, percentage of implausible values for height, weight, BMI; preference for terminal digit of weight and height, and normality of distributions.

**Results:**

The database of pregnant women made available for analysis included 8,046,608 records and 1,174,115 women. The percentage of incompleteness and zero values was low (<1%) in all original variables of the system. There are more records at the end of pregnancy. For the four original variables of interest in the database (weight, height, SBP, DBP), there is a clear preference for the terminal digit. The variables of interest did not present an approximately normal distribution during the evaluated period.

**Conclusion:**

The quality analysis showed the need for improving the standardization of information collection and recording, the rounding of measurements and the need for encouraging pregnant women to start prenatal care as soon as possible, in such a way that it is important to invest in data quality, through educational resources for professionals who work in health care.

## RESUMO

**Objetivo:**

Analisar a qualidade dos dados coletados no acompanhamento pré-natal registrados no Sistema Integrado de Gestão da Assistência à Saúde (SIGA) da Secretaria Municipal de Saúde de São Paulo de 2012 a 2020.

**Métodos:**

Estudo descritivo utilizando dados do SIGA e as variáveis: altura materna (cm), peso (kg) medido ao longo da gestação, idade gestacional na consulta pré-natal, pressão arterial (em mmHg) sistólica (PAS) e diastólica (PAD), e índice de massa corporal (IMC) no início da gestação (até 8 semanas). A análise da qualidade foi realizada por meio do cálculo dos indicadores: percentual de incompletude e valores zero de todas as variáveis estudadas, percentual de valores implausíveis de estatura, peso, IMC; preferência por dígito terminal do peso e estatura, e normalidade das distribuições.

**Resultados:**

Base de dados de gestantes disponibilizada para análise incluía 8.046.608 registros e 1.174.115 mulheres. O percentual de incompletude e valores zeros foi baixo (<1%) em todas as variáveis originais do sistema. Existe maior número de registros ao final da gestação. Para as quatro variáveis de interesse originais do banco de dados (peso, altura, PAS, PAD), existe clara preferência por dígito terminal. As variáveis de interesse não apresentaram distribuição aproximadamente normal durante o período avaliado.

**Conclusão:**

A análise da qualidade mostrou necessidade de melhoria na padronização da coleta e do registro das informações, no arredondamento das medidas e na necessidade de incentivar as gestantes a iniciar o pré-natal o quanto antes; por isso, é importante investir na qualidade do dado, por meio de recursos educativos para profissionais que atuam na assistência.

## INTRODUCTION

Recording administrative data collected in prenatal care and childbirth can generate a large volume of information about the provided services. These data, when adequately collected, recorded and processed, can support managers in decision-making related to healthcare planning and surveillance^1,2^.

The exploration of secondary databases for administrative use, mainly from health information systems, can also substantially contribute to the knowledge of local needs. However, the use of these databases must be preceded by understanding their limits, such as the lack of standardization for collection and errors resulting from the lack of consistency in data entry into the system. Furthermore, it is necessary to know the population coverage to define its representativeness. There are problems in both the quality and type of information obtained; therefore, assessing the completeness and quality of these data is necessary^3^.

In the municipality of São Paulo, a system for scheduling appointments, controlling the waiting list and the number of appointments per unit during prenatal care was created in 2004. The Integrated Health Care Management System (*Sistema Integrado de Gestão da Assistência à Saúde* – SIGA) assists managers in organizing the referral and counter-referral system of healthcare services and hospitals that will perform deliveries. In addition, it enables to characterize pregnant women seen in the municipality; however, its use has only been administrative in nature, and there has been little or no use of the data for an epidemiological diagnosis.

Statistical analysis of datasets often encounters challenges such as incompleteness, zero values, and implausibility. Incompleteness refers to the absence of data in certain observations, which may compromise the integrity of the analyses. Zero values can indicate different scenarios, from the actual absence of occurrences to reporting issues. Implausibility addresses values that are outside the possible range or that do not make sense in the context of the study. To evaluate the dissimilarity between data sets, the index of dissimilarity is used, which quantifies the differences between data patterns^4^. Studies^5-8^ show that inconsistencies found in databases can harm the quality of the information made available and the comprehensive assessment of individuals.

SIGA was implemented in other municipalities in Brazil; nevertheless, to date, there are no quality analyses of the data generated in the municipality of São Paulo. Therefore, this study aimed to analyze the quality of data collected from prenatal care and recorded in the SIGA of the Municipal Department of Health of the state of São Paulo, Brazil, between 2012 and 2020.

## METHODS

This is a descriptive study using data from pregnant women registered in SIGA between 2012 and 2020. The data are collected during routine prenatal care and entered into the system by professionals from the Healthcare Services (*Unidades Básicas de Saúde* – UBS). Overall, administrative employees type and collect information about user's identification and appointment scheduling, and technicians (physicians, nurses, nutritionists) collect and type other information regarding the provided care, for example, weight, height, blood pressure, among others.

The resident population of the city of São Paulo, in 2020, was estimated by the SEADE Foundation (*Sistema Estadual de Análise de Dados* – State System for Data Analysis) in 11,869,660 inhabitants^9^. In the city's Brazilian Unified Health System (SUS), the units responsible for primary health care totaled 469^10^ in 2020, with 85% UBS or Family Healthcare Services and others, Healthcare Services/Outpatient Medical Assistance (UBS/AMA). All of these units feed SIGA with data on the provision of care in primary health care and specialized outpatient clinics. In the present study, data from one of the system's modules, namely SIGA/Mãe paulistana [Mother from São Paulo], was analyzed, in which data from pregnant women who undergo prenatal care at UBS are entered.

Among the SIGA variables, the following were considered for this analysis: maternal height (cm), weight (kg) measured throughout pregnancy, gestational age (in weeks) at the prenatal consultation, systolic blood pressure (SBP) and diastolic blood pressure (DBP), in mmHg. Two derived variables were created from these: the weight measured at the beginning of pregnancy (up to 8 weeks), as this is a variable that allows the calculation of gestational weight gain, and the body mass index (BMI) at the beginning of pregnancy (up to 8 weeks), calculated by dividing the weight measured up to 8 weeks by the maternal height (in meters) squared. BMI is used to diagnose maternal nutritional status at the beginning of pregnancy^11^.

The analysis of the quality of the database was carried out by calculating the following indicators: percentage of incompleteness and zero values, percentage of implausible values, preference for the terminal digit, and normality of distributions. These indicators were adapted from indicators used to assess the quality of anthropometric data on children^12^.

Incompleteness was defined as the percentage of information not filled in or zero values^13^. The degree of incompleteness was defined according to the cutoff points proposed by Romero and Cunha^14^: incompleteness below 5% was considered excellent; from 5 to 9.9%, good; from 10 to 19.9%, regular; from 20 to 49.9%, poor; and 50% or more, very poor.

Implausible values were defined differently for each variable of interest, and, whenever possible, external references were used. For maternal age, values below 10 and above 55 years were considered implausible; this criterion was used considering the age profile of the mother of live births in Brazil^15^.

For height and BMI, values below −5 Z-score and above 5 Z-score were considered implausible, and for weight, values below −6 Z-score and above 6 Z-score^16^. The World Health Organization (WHO)^17^ curves were used to define the cutoff points.

For the purpose of a cutoff point for the distribution of scores and greater similarity between the values of adults and adolescents, the same curve was standardized, considering the age of 19 years for all adult pregnant women, as there is no distribution curve for implausible values for those over 19 years of age.

For SBP and DBP, Z-scores outside the range of −6 to 6 of the sampling distribution for each trimester of pregnancy were considered implausible values.

The preference for terminal digits was evaluated using graphs and by calculating the index of dissimilarity, obtained using the formula (Equation 1):


(1) Index of dissimilarity=∑abs (observed percentage−expected percentage)/2

The index of dissimilarity can be interpreted as the percentage of values that would need to be redistributed so that the distribution of the final digits of the variable of interest is uniform (that is, without rounding). Values above 20% indicate preference for terminal digit and rounding to 0 or 5^12^.

The kurtosis coefficient indicates the thickness of the tails of the distribution compared to the normal distribution. A normal distribution has a kurtosis coefficient equal to zero. Positive values of kurtosis indicate that the tails of the distribution are shorter than those of the normal distribution, and negative values indicate longer tails^18^. All analyses were performed in the *R* software version 4.1.0^19^.

This study is part of the research project entitled *Como tornar as intervenções no parto e seus desfechos mais visíveis aos sistemas de informação?* [“How to make childbirth interventions and their outcomes more visible to information systems?”] (project with funding already approved in the Call for Data Science for Maternal and Child Health CNPq/Bill & Melinda Gates Foundation/2020/2022), approved by the Research Ethics Committee of the Municipal Department of Health of São Paulo, under number 4.829.5.

## RESULTS

The SIGA database of pregnant women made available for analysis included 8,046,608 records and 1,174,115 women from 2012 to 2020. The number of records varied by year of follow-up, with an increase in the number of consultations registered in the system from 2015 onward and a small reduction in 2018. This number remained stable between the months and years of 2018 to 2020 (Supplementary Figure 1).

The percentage of incompleteness and zero values was low (<1%) in all original variables of the system, deemed as excellent^14^. Higher incompleteness values were observed for the variables weight and BMI at the beginning of pregnancy (75.69%) in 2012, considered very poor^14^. We observed no variations in these percentages over the years. For the variables weight and BMI at the beginning of pregnancy, the percentage of incompleteness was high throughout the period, exceeding 60% by 2017. A tendency to reduce the percentage of incompleteness in these two variables over time was observed (for both variables, 75.69% in 2012 versus 49.93% in 2020). All registered women presented at least one weight and one height record. The percentage of implausible Z-scores was also low across all variables of interest (<0.5%), with relative stability over time (Table 1 and Supplementary Figure 2).

**Table 1:** Absolute and relative frequency of incompleteness values, zero values and implausible Z-scores in the variables of interest in the pregnant women module of the Integrated Health Care Management System of the Municipal Department of Health of São Paulo, municipality of São Paulo, 2012 to 2020.

Variable*		Total	2012	2013	2014	2015	2014	2017	2018	2017	2020
Gestational age											
	Total records	8,047,175	795,244	796,098	831,501	865,680	940,140	978,259	967,233	950,203	601,716
	Incompleteness	0 (0)	0 (0)	0 (0)	0 (0)	0 (0)	0 (0)	0 (0)	0 (0)	0 (0)	0 (0)
	Zero values	567 (0.007)	27 (0.0034)	53 (0.0066)	58 (0.0072)	48 (0.0056)	66 (0.0072)	62 (0.0062)	100 (0.0104)	79 (0.0082)	74 (0.0077)
Maternal height											
	Total records	8,047,175	795,244	796,098	831,501	865,680	940,140	978,259	967,233	950,203	601,716
	Incompleteness	0 (0)	0 (0)	0 (0)	0 (0)	0 (0)	0 (0)	0 (0)	0 (0)	0 (0)	0 (0)
	Zero values	0 (0)	0 (0)	0 (0)	0 (0)	0 (0)	0 (0)	0 (0)	0 (0)	0 (0)	0 (0)
	Implausible Z-scores	1,885 (0.023)	165 (0.021)	197 (0.025)	136 (0.017)	207 (0.024)	198 (0.022)	254 (0.026)	214 (0.022)	288 (0.03)	49 (0.031)
Weight at the beginning of pregnancy (up to 8 weeks)											
	Total records	1,174,088	121,510	122,745	125,659	124,753	125,971	130,760	127,892	121,951	107,284
	Incompleteness	793,239 (67.56)	91,969 (75.69)	92,249 (75.15)	92,141 (73.33)	88,484 (70.93)	81,344 (64.57)	80,310 (61.42)	77,461 (60.57)	71,483 (58.62)	53,563 (49.93)
	Zero values	0 (0)	0 (0)	0 (0)	0 (0)	0 (0)	0 (0)	0 (0)	0 (0)	0 (0)	0 (0)
	Implausible Z-scores	33 (0.003)	2 (0.007)	0 (0)	2 (0.006)	4 (0.011)	1 (0.002)	5 (0.01)	3 (0.006)	9 (0.018)	0 (0)
BMI at the beginning of pregnancy											
	Total records	1,174,088	121,510	122,745	125,659	124,753	125,971	130,760	127,892	121,951	107,284
	Incompleteness	793,247 (67.56)	91,970 (75.69)	92,249 (75.15)	92,141 (73.33)	88,485 (70.93)	81,345 (64.57)	80,312 (61.42)	77,464 (60.57)	71,483 (58.62)	53,563 (49.93)
	Zero values	0 (0)	0 (0)	0 (0)	0 (0)	0 (0)	0 (0)	0 (0)	0 (0)	0 (0)	0 (0)
	Implausible Z-scores	776 (0.066)	36 (0.12)	35 (0.114)	48 (0.143)	57 (0.157)	73 (0.162)	111 (0.219)	101 (0.201)	135 (0.268)	26 (0.321)
Weight during pregnancy (distribution by gestational week)											
	Total records	8,047,175	795,244	796,098	831,501	865,680	940,140	978,259	967,233	950,203	601,716
	Incompleteness	0 (0)	0 (0)	0 (0)	0 (0)	0 (0)	0 (0)	0 (0)	0 (0)	0 (0)	0 (0)
	Zero values	5 (0.0001)	0 (0)	1 (0.0001)	3 (0.0004)	0 (0)	0 (0)	0 (0)	0 (0)	1 (0.0001)	0 (0)
	Implausible Z-scores	1,531 (0.019)	87 (0.011)	110 (0.014)	102 (0.013)	112 (0.013)	183 (0.02)	228 (0.023)	213 (0.022)	239 (0.025)	41 (0.026)
Diastolic blood pressure											
	Total records	8,047,175	795,244	796,098	831,501	865,680	940,140	978,259	967,233	950,203	601,716
	Incompleteness	0 (0)	0 (0)	0 (0)	0 (0)	0 (0)	0 (0)	0 (0)	0 (0)	0 (0)	0 (0)
	Zero values	1,524 (0.0189)	41 (0.0051)	171 (0.0213)	328 (0.0405)	183 (0.0215)	115 (0.0126)	140 (0.0141)	206 (0.0215)	176 (0.0184)	164 (0.0171)
	Implausible Z-scores	802 (0.01)	15 (0.002)	42 (0.005)	77 (0.01)	79 (0.009)	75 (0.008)	109 (0.011)	115 (0.012)	152 (0.016)	23 (0.015)
Systolic blood pressure											
	Total records	8,047,175	795,244	796,098	831,501	865,680	940,140	978,259	967,233	950,203	601,716
	Incompleteness	0 (0)	0 (0)	0 (0)	0 (0)	0 (0)	0 (0)	0 (0)	0 (0)	0 (0)	0 (0)
	Zero values	772 (0.0096)	13 (0.0016)	115 (0.0144)	270 (0,0334)	126 (0.0148)	42 (0.0046)	26 (0.0026)	85 (0.0089)	41 (0.0043)	54 (0.0056)
	Implausible Z-scores	3,148 (0.039)	58 (0.007)	153 (0.019)	312 (0.039)	263 (0.031)	212 (0.023)	405 (0.041)	474 (0.049)	604 (0.063)	96 (0.061)

BMI: body mass index

* other variables in the database did not present incompleteness or zeros.

Regarding the number of records (consultations) according to gestational age, we observed that the largest volume occurs at the end of pregnancy, especially in the last trimester (from 27 weeks onward) (Supplementary Figure 3). This pattern is repeated for all evaluated years, and 3,885,165 (48.28%) records occurred between 27 and 40 weeks of gestation, in the period between 2012 and 2020.

**Figure 1:**
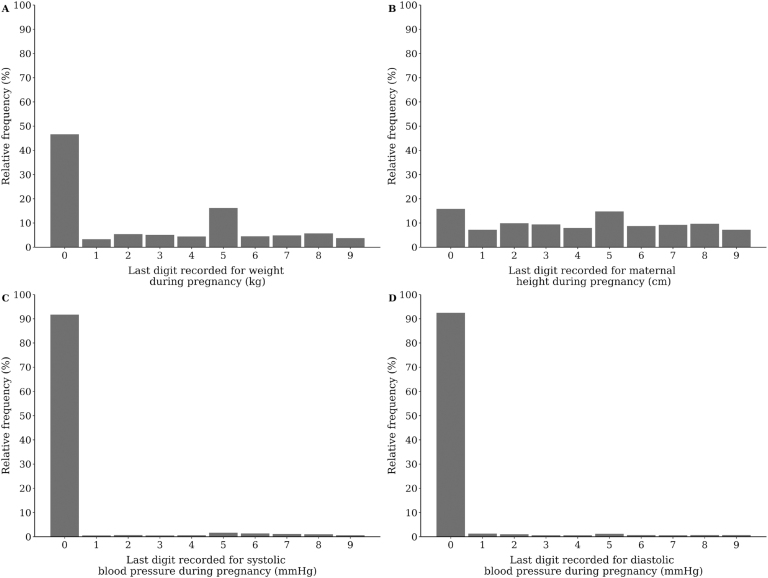
Identification of preference for terminal digit in anthropometric variables: (A) weight during pregnancy (kg); (B) maternal height (m); (C) systolic blood pressure (mmHg); and (D) diastolic blood pressure (mmHg) from the pregnant women module of the Integrated Health Care Management System of the Municipal Department of Health of São Paulo, 2012 to 2020.

For weight, height, SBP and DBP, there is a clear preference for terminal digit, that is, the measured values are clearly rounded to 0 or 5 (Figure 1). This rounding pattern is confirmed by the values observed for the index of dissimilarity, which is greater than 20% for weight, SBP and DBP, in all evaluated years (Table 2). For maternal height, despite the frequency of values 0 and 5 being higher than the frequency observed for other terminal digits (Figure 1), the values for the index of dissimilarity remained close to 10% in the period (Table 2).

**Table 2:** Index of dissimilarity for the variables of interest in the pregnant women module of the Integrated Health Care Management System, municipality of São Paulo, 2012 to 2020.

Variable of interest	Total	2012	2013	2014	2015	2014	2017	2018	2017	2020
Weight during pregnancy	42.81	44.22	44.28	43.64	43.17	43.45	42.61	43.37	41.45	39.76
Maternal height	10.58	11.82	10.97	10.83	10.40	10.10	9.90	10.33	10.74	10.59
SBP	82.44	86.67	85.50	84.38	84.47	83.92	82.40	81.00	78.43	76.97
DBP	81.75	86.37	85.27	84.11	83.73	83.25	81.67	80.07	77.49	75.80

SBP: systolic blood pressure; DBP: diastolic blood pressure.

The variables of interest did not present an approximately normal distribution during the evaluated period. Overall, the variables presented standard deviation values close to 1, with the lowest value observed for DBP in 2014 (0.752) and the highest for BMI at the beginning of pregnancy, in 2020 (1.223). SBP and DBP showed left asymmetry (negative) throughout the period, except for SBP in 2012. The other variables presented an asymmetry coefficient close to zero. All variables presented a kurtosis coefficient above 0; the lowest value was 2.95 (BMI at the beginning of pregnancy) and the highest was 17.85 (DBP), which indicates leptokurtic distribution (Table 3).

**Table 3:** Standard deviation, asymmetry measures and kurtosis of Z-scores of anthropometric variables of pregnant women in the municipality of São Paulo, 2012 to 2020.

Variable	Statistics	Total	2012	2013	2014	2015	2014	2017	2018	2017	2020
Maternal height	Standard deviation	0.996	0.986	0.983	0.986	0.990	0.990	0.998	1.001	1.006	1.002
	Asymmetry	0.11	0.12	0.11	0.12	0.12	0.12	0.12	0.12	0.12	0.12
	Kurtosis	3.28	3.35	3.34	3.30	3.30	3.25	3.24	3.29	3.27	3.23
Weight at the beginning of pregnancy (up to 8 weeks)	Standard deviation	0.988	0.907	0.929	0.935	0.947	0.969	0.991	0.997	1.022	1.052
	Asymmetry	0.98	1.09	1.05	1.05	1.01	0.96	0.97	0.93	0.93	0.89
	Kurtosis	4.60	4.98	4.85	4.89	4.77	4.58	4.61	4.39	4.40	4.37
BMI at the beginning of pregnancy	Standard deviation	1.196	1.145	1.163	1.165	1.174	1.188	1.191	1.203	1.213	1.223
	Asymmetry	0.03	0.14	0.11	0.09	0.06	0.02	0.04	-0.01	-0.03	-0.08
	Kurtosis	2.95	3.02	2.97	3.01	2.99	2.95	2.97	2.96	2.95	2.95
Weight during pregnancy (distribution by trimester)	Standard deviation	0.944	0.921	0.923	0.927	0.932	0.933	0.948	0.945	0.954	0.964
	Asymmetry	0.45	0.45	0.44	0.44	0.45	0.44	0.44	0.44	0.44	0.45
	Kurtosis	4.37	4.19	4.20	4.34	4.48	4.36	4.37	4.38	4.45	4.35
Weight during pregnancy (distribution by gestational week)	Standard deviation	0.950	0.930	0.930	0.934	0.939	0.939	0.953	0.950	0.959	0.968
	Asymmetry	0.45	0.45	0.44	0.44	0.44	0.44	0.44	0.44	0.48	0.45
	Kurtosis	4.37	4.14	4.19	4.29	4.48	4.37	4.39	4.44	4.43	4.35
Systolic blood pressure	Standard deviation	0.941	0.778	0.849	0.929	0.912	0.890	0.922	0.963	0.986	1.038
	Asymmetry	-1.92	-0.71	-1.33	-1.86	-1.80	-1.64	-1.83	-2.00	-2.08	-2.22
	Kurtosis	13.01	9.71	11.84	12.94	13.01	12.68	12.92	13.08	12.99	12.75
Diastolic blood pressure	Standard deviation	0.967	0.752	0.859	1.011	0.928	0.878	0.948	0.977	1.051	1.060
	Asymmetry	-2.65	-1.33	-2.20	-2.70	-2.52	-2.39	-2.62	-2.67	-2.67	-2.80
	Kurtosis	17.10	14.49	17.27	16.38	17.32	17.85	17.32	17.11	15.95	16.01

BMI: body mass index.

## DISCUSSION

Studies on data quality in developing countries are limited^20^. This study was the pioneer in qualifying the data collected during prenatal care and recorded in SIGA. There is no consensus regarding data quality assessment criteria^21^. In this study, we used percentage of incompleteness and zero values, percentage of implausible values, preference for terminal digit, and normality of distributions.

For the percentage of incompleteness, values classified as very poor were found in the variables initial weight and initial BMI throughout the analyzed period. High incompleteness values were also found in the study by Romero and Cunha^14^, who evaluated the quality of socioeconomic and demographic information, by Federative Unit of the Brazilian Mortality Information System (*Sistema de Informações sobre Mortalidade* – SIM). However, the percentage of incompleteness and zero values was low in all of the system's original variables, considering, in this regard, good quality data. A database must be complete and reliable regarding its records^22^. When these data are inconsistent, the reliability of the information is compromised and false diagnoses about the health situation can be established^23^.

Regarding the preference for terminal digit, in this study, there is a preference for the terminal digits 0 or 5 in the variables weight, height, SBP and DBP, similar to the National Survey of Food and Child Nutrition (*Estudo Nacional de Alimentação e Nutrição Infantil* – ENANI) of 2019^16^, which found a preference for terminal digit for weight and height.

We found low percentages of implausible Z-score values in all studied variables (<0.5%), as can be seen in the Supplementary Figure 2, remaining within the internationally recommended range (1%)^12^.

According to the Brazilian Ministry of Health, pregnant women must have at least six prenatal consultations, preferably one in the first trimester, two in the second trimester, and three in the third trimester of pregnancy^24^. In this study, we show an increase in the number of prenatal consultation records over the period, but it is not possible to know whether the number of consultations actually increased or whether more records were made in the system. The low percentage of consultation records at the beginning of pregnancy is similar to the findings of Domingues et al.^25^ and Kac et al.^11^, who show a high proportion of Brazilian pregnant women starting prenatal care after 12 weeks.

Weight measurement must be carried out at all prenatal consultations, and height must be measured at least during the first consultation of pregnant women^26^. In the SIGA data, we can observe that all women have at least one weight and one height record during pregnancy. However, when evaluating weight information collected at the beginning of pregnancy (up to 8 weeks), the percentage of incompleteness is high throughout the evaluated period. The absence of weight recording at the beginning of pregnancy is reflected in the BMI during the period, and represents an important limitation for characterizing the nutritional status of these individuals and using indicators recommended by the Ministry of Health such as weight gain during pregnancy. In this case, the use of weight at the time of pregnancy diagnosis could be recommended, as long as pregnant women were instructed to record their weight at that time.

Pre-pregnancy BMI and weight gain during pregnancy are related to fetal and neonatal development as well as obstetric outcomes^27^. Taking this into consideration, the importance of collecting quality information during the prenatal period is highlighted, contributing to more effective monitoring and obtaining more accurate and valuable future indicators, aiming to improve public policies with specific nutritional guidance for this group, improving pregnant women's access to less processed foods, with lower fat, sodium and sugar content, as well as greater amounts of vitamins and minerals^28,29^.

We verified some limitations of the system (SIGA), such as the gestational age being in weeks and not in days, which reduces the precision of this datum, also as a result of non-standard rounding^30^. Another limitation of the system was the lack of standardization of collection. In the municipality of São Paulo, the process of collecting prenatal data, as well as other data, takes place in more than 469^10^ units, and these data are entered by professionals in charge in each unit. The in-service training of these professionals does not always meet their needs, sometimes resulting in inconsistent, non-standardized, and incomplete data^31^. In the case of the weight and height of pregnant women, both the measurement techniques and the recording may be incorrect, hence the importance of providing instruments to the involved professionals and returning the data for analysis to the units responsible for collecting and typing them.

This study is the first to use SIGA data, which makes it unprecedented and extremely important for managers’ knowledge of data from maternity wards and UBS in their territory. Despite this important positive aspect, the present study have some limitations, such as the use of quality indicators adapted from those used for children, as there are no specific indicators for pregnant women.

The analyses carried out in this study enabled to show the need for an improvement in the standardization of the collection and recording of information, for example, stipulating clear rules for rounding measurements for greater precision. Another important aspect is the need to encourage pregnant women to start prenatal care as soon as possible, be properly informed and actively participate in monitoring their personal health indicators (such as weight, blood glucose and blood pressure) as well as monitoring and improving the quality of these data in service records.

Improving the SIGA database data would result in an improvement in data quality, enabling to expand its use for calculating indicators and to monitor public policies aimed at pregnant women. In this sense, it is extremely important to invest in the quality of data, through educational resources for those responsible for filling in the data^32^. The regular dissemination and use of SIGA prenatal information should be encouraged, as it is valuable for epidemiological analyses and can contribute to improving its quality^1^. Moreover, we highlight the importance of evaluating the quality of the SIGA database, as it can be linked, for example, with the live birth database, SINASC (*Sistema de Informação sobre Nascidos Vivos* – Brazilian Live Birth Information System), to analyze outcomes in relation to the baby, the mother, and the delivery condition.
