# Supplementary material for: Analysis of the quality of prenatal data of pregnant women attended at Healthcare Services in the city of São Paulo between 2012 and 2020
Source: Rev Bras Epidemiol. 2023 Nov 13;26:e230051. doi: 10.1590/1980-549720230051 (PMC10662653; doi:10.1590/1980-549720230051)
Supplement: Supplementary file 1 [file 1980-5497-rbepid-26-e230051-Suppl01.pdf]

Figura A

Figura B

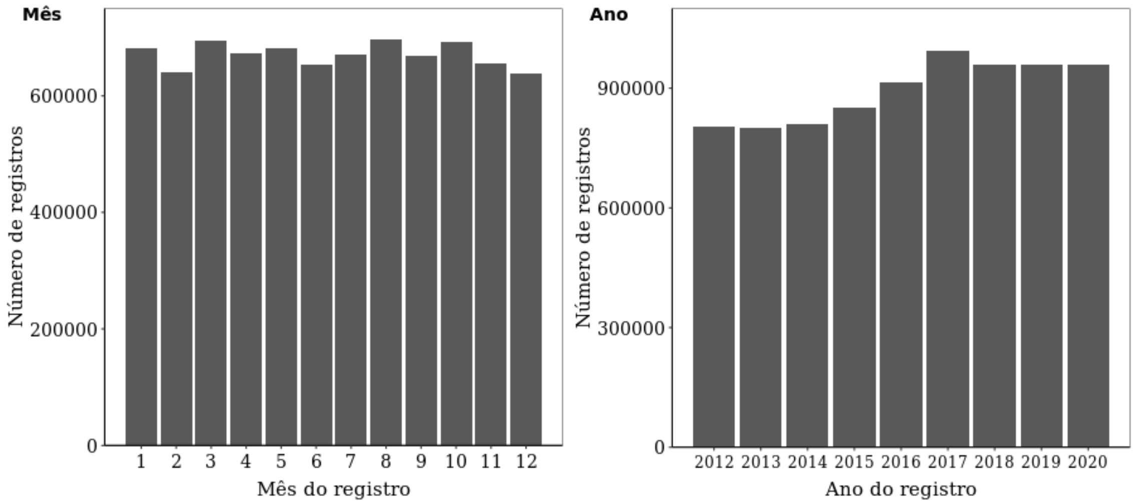

Figura suplementar 1. Distribuição da quantidade de consultas de acordo com os meses do ano e com o ano de acompanhamento da gestante.

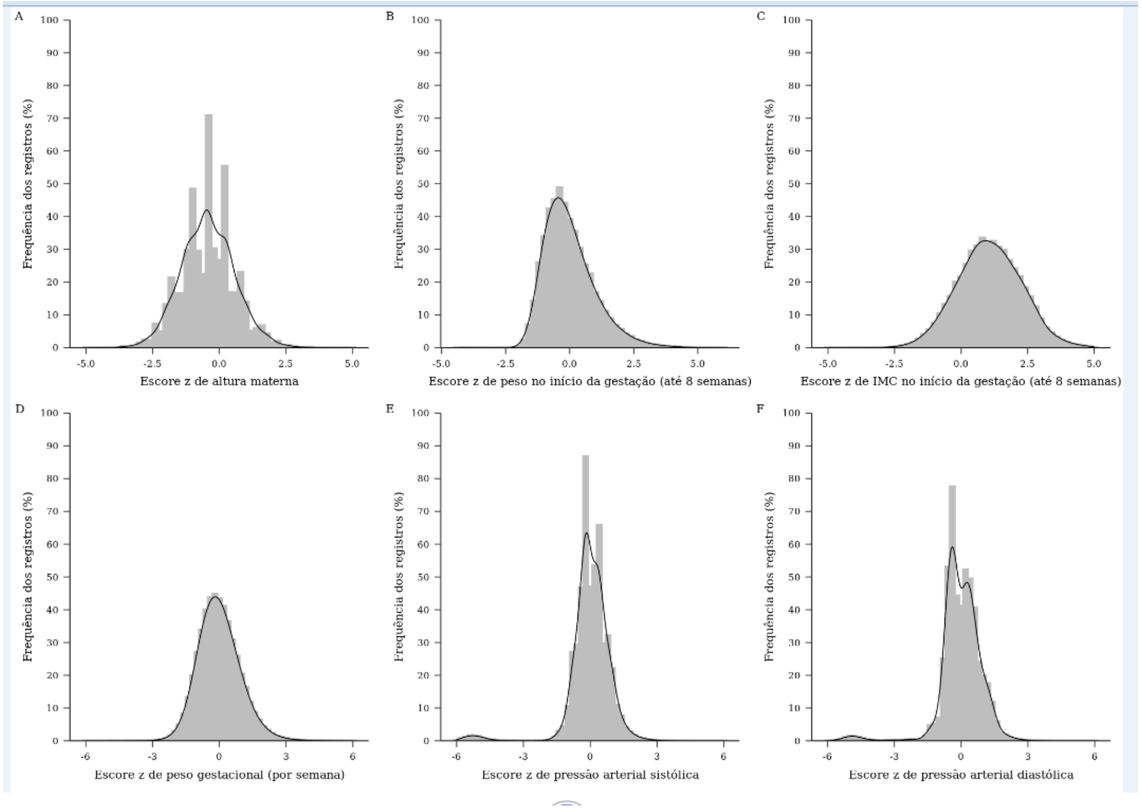

Figura suplementar 2. Histogramas para os escores z das variáveis antropométricas: A. Altura materna; B. Peso no início da gestação; C. IMC no início da gestação; D. Peso durante a gestação (por trimestre); E. Peso durante a gestação (por semana); F. Pressão arterial diastólica; G. Pressão arterial sistólica.

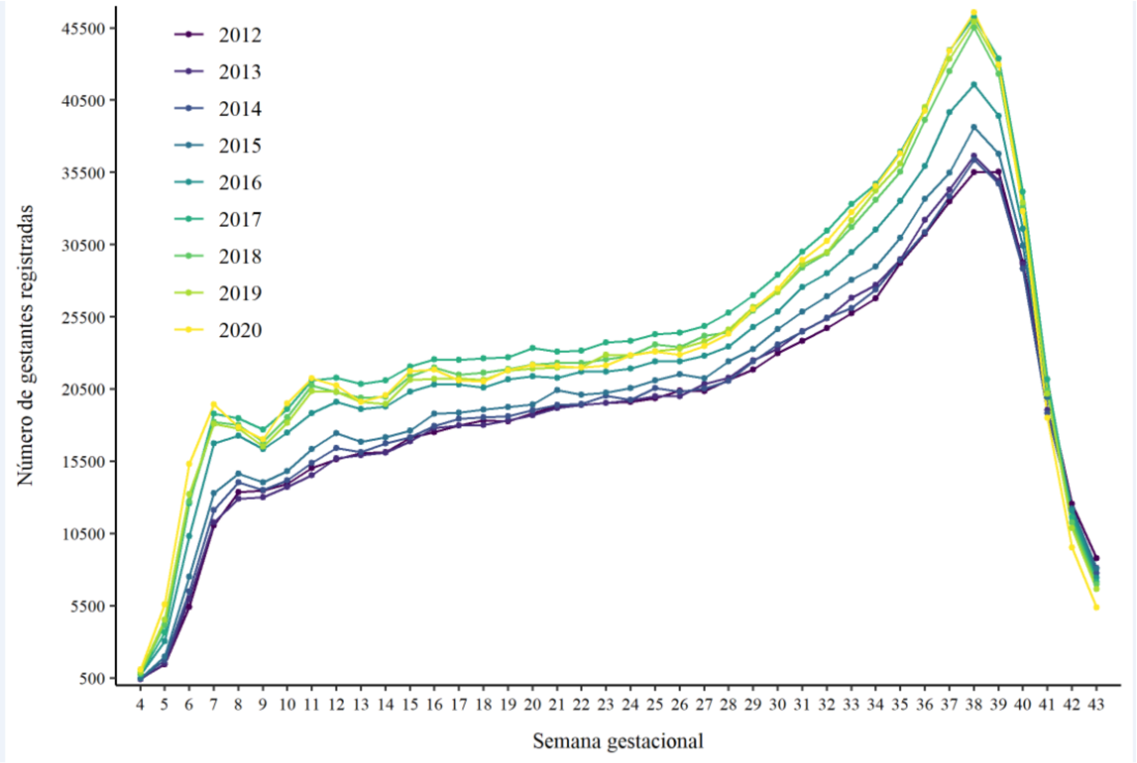

Figura suplementar 3. Distribuição da quantidade de registros (consultas) por idade gestacional em semanas.
